# ASO Author Reflections: Precision in Practice: Breast Surgeons as Catalysts for Quality Improvement

**DOI:** 10.1245/s10434-025-18686-8

**Published:** 2025-11-05

**Authors:** Sara Grossi, Kandice Ludwig

**Affiliations:** 1 University of California San Diego School of Medicine, San Diego, CA USA; 2 Indiana University School of Medicine, Indianapolis, IN USA

## Past

The American College of Surgeons (ACS) has a long history of quality improvement (QI) initiatives, and adherence to strict quality standards and established guidelines are utilized to maintain accreditations in specialty care. The ACS developed standards of care for cancer and instituted surveys to assess compliance, laying the foundation for the modern-day Commission on Cancer and the National Accreditation Program for Breast Centers. These accreditation programs have developed evidence-based standards to improve patient care and outcomes in collaboration with subject matter experts.^1^ These quality standards have historically been utilized by the Centers for Medicare and Medicaid Services (CMS) to measure or quantify healthcare processes, outcomes, patient perceptions, and organizational structure that are associated with the ability to provide high-quality healthcare.^2^

Engagement in QI by the breast surgeon has been and continues to be paramount for adherence and accreditation in specialty care. The breast surgeon is uniquely positioned to assess and implement changes in measurable outcomes from diagnosis to treatment and is often tasked with initiating these QI projects. While many surgeons are comfortable interpreting data to make clinical decisions and regularly incorporate evidence-based guidelines into their practices, most lack formal training in quality.

## Present

The Patient Safety and Quality (PSQ) Committee of the American Society of Breast Surgeons created a workgroup to develop a practical and easily accessible “how to” guide for breast surgeons to initiate and navigate QI projects. First, a search of peer-reviewed publications and other relevant websites pertaining to quality in healthcare, cancer, and surgery was performed to identify key elements of the quality process. In addition, examples of QI projects from their respective home institutions were reviewed. All of the data sources were combined to establish nine key principles of the quality improvement study process. The guide also provides ideas of possible projects, sources of data, and granular examples specific to breast surgery. ^3^

## Future

The landscape of breast surgery is continually evolving, with new surgical techniques, developing technology, and changes in management of women with breast cancer. A combined effort between healthcare staff and other stakeholders should be made to assure that these changes improve efficiency, expand access to care, improve outcomes, and reduce costs.^4^ The breast surgeon is integral as a catalyst for these efforts. The surgeon may utilize an array of methods to identify problems, implement changes on a smaller scale, monitor results, and decide whether wider implementation can be accomplished. Moreover, both clinicians and patients have an ethical responsibility to participate in QI for the shared expectation and future development of high-quality healthcare that is safe, effective, patient-centered, timely, equitable, and efficient.^5^
